# How Does Supportive Leadership Impact the Safety Behaviors of the New Generation of Construction Workers?

**DOI:** 10.3390/bs15020110

**Published:** 2025-01-22

**Authors:** Libing Cui, Junwen Mo

**Affiliations:** Department of Engineering Management, School of Civil Engineering, Lanzhou Jiaotong University, Lanzhou 730070, China; 11200226@stu.lzjtu.edu.cn

**Keywords:** supportive leadership, new-generation construction workers, safety behaviors, safety self-efficacy, team safety climate

## Abstract

Numerous other scholars have, from an organizational perspective, underscored that leadership styles, such as transformational and transactional leadership, are pivotal antecedents to the safety behaviors of construction workers. However, there is a lack of research exploring the relationship between leadership styles and the safety behaviors of this specific group of new-generation construction workers at the individual level. Relying upon social exchange theory, social cognitive theory, and contingency theory, this research explores the impact of supportive leadership—characterized by care and support—on the safety behaviors (safety compliance and safety participation) of new-generation construction workers. A comprehensive approach combining questionnaire surveys, confirmatory factor analysis, correlation tests, and linear regression was adopted. The results demonstrate that supportive leadership has a significant positive influence on the safety behaviors of new-generation construction workers. Moreover, safety self-efficacy partially mediates this relationship. Additionally, a positive team safety climate enhances the effect of supportive leadership on safety participation. This study provides a theoretical foundation for optimizing leadership styles to improve safety behaviors among new-generation construction workers and offers new insights into the nuanced application of leadership styles in construction safety management.

## 1. Introduction

The “14th Five-Year Plan” has set an inspiring trajectory for the high-quality development of China’s construction industry, showcasing its robust vitality and significant strength. Underpinned by strategies such as the steady advancement of new urbanization and the reinforcement and upgrading of infrastructure, the construction industry resembles a colossal ship navigating through turbulent waves, charting a steadfast course and seamlessly integrating into the broader wave of the nation’s modernization drive (MOHURD, 2022). However, despite the apparent prosperity, the frequent occurrence of construction safety accidents has caused significant losses to human lives, properties, families, and the reputations of companies (Estudillo et al., 2024). Statistics reveal that among the numerous factors contributing to safety accidents, the majority are linked to human factors, with over 80% of workplace accidents attributed to employees’ unsafe behaviors (Fang et al., 2015; Patel & Jha, 2015). Notably, as time progresses, the new generation of construction workers—those born in the 1980s and later—has gradually become the primary workforce in the construction industry (Ni et al., 2023; Zhao et al., 2024). Compared with the older generation of construction workers, this generation is generally more educated, self-aware, and has more diverse expectations regarding their work environment and career development. They seek a better work-life balance. However, the construction industry is notorious for its harsh and hazardous working conditions, with limited opportunities for career advancement, which fail to meet the diverse needs of these younger workers. Furthermore, the industry often demands long hours of intense labor, leading to a work-life imbalance. This gap makes it difficult for workers to develop a sense of belonging to the profession (Lu et al., 2023; Zhang, 2018). Moreover, the new generation of construction workers is regularly exposed to extreme conditions—severe dust, noise pollution, and high safety risks at construction sites—leading to the erosion of both their physical and mental health. The grueling work schedules rob them of essential rest, leaving them physically and mentally drained. The limited career prospects create a sense of hopelessness about their future, while social isolation and financial pressures contribute to heightened anxiety and depression. These challenges cause many to fall into negative emotional states such as anxiety and depression. Faced with complex problems and harsh criticism, they may experience emotional outbursts, engage in passive work behaviors, or become careless in their tasks, ultimately leading to unsafe behaviors (Sámano-Ríos et al., 2019; Yu et al., 2022; Zhou et al., 2024). Consequently, addressing how to enhance the safety behaviors of the new generation of construction workers has become a pressing scientific issue requiring immediate attention.

Corporate leadership refers to a leader’s ability and influence to guide a team toward achieving goals, driving organizational growth, and addressing challenges within the corporate environment. It encompasses various dimensions, including decision-making, motivational strategies, and communication styles. Previous studies have demonstrated that leadership styles—including transformational leadership (Afsar et al., 2019; Khan & Khan, 2022; Lee & Chon, 2021), transactional leadership (Bian et al., 2019; Faraz et al., 2018), and supportive leadership (Elsaied, 2019; Wei et al., 2013), among others—play a critical role in shaping employees’ behaviors. In essence, leadership style serves as a significant factor influencing employee behavior. For instance, safety transformational leadership focuses on influencing employees’ safety behaviors by stimulating their higher-level needs and shaping the organizational vision. It pays more attention to the guidance and shaping at the ideological level and often endeavors to create a grand atmosphere oriented towards the overall safety mission of the organization (Hoffmeister et al., 2014; B.-J. Kim & Jung, 2019; D. Wang et al., 2023). Transactional leadership, in order to achieve the organization’s safety goals, emphasizes clear task assignments and a reward-punishment system, establishing “contracts” for safety standards. Employees who meet these standards will receive rewards from the organization, while those who violate them will be punished (Bian et al., 2019; Faraz et al., 2018). Both safety transformational leadership and transactional leadership aim to regulate employees’ behaviors on the organizational level under the premise of achieving the organization’s safety objectives. However, the difference is that the supportive leadership style is more in line with the construction industry’s pursuit of people-oriented and high-quality safety management. It focuses more on the care and support for the individual level of the new-generation construction workers (Elsaied, 2019; Wei et al., 2013). On the one hand, supportive leadership emphasizes caring about employees’ personal feelings and needs, providing them with work guidance and psychological support, which meets the expectations of the new-generation construction workers, making them feel respected and cared for, so that they are more willing to actively abide by safety behavior norms. In contrast, transformational leadership focuses on motivating employees to pursue lofty goals, and transactional leadership mainly focuses on the exchange of tasks and rewards, being less targeted at meeting the emotional and psychological needs of the new-generation construction workers. On the other hand, supportive leadership can pay attention to workers’ working conditions, such as fatigue levels, and offer timely support to reduce potential safety hazards caused by factors such as fatigue. Transformational leadership, which emphasizes vision incentives, may cause employees to focus more on performance and overlook some basic safety details (Afsar et al., 2019; Lee & Chon, 2021). Transactional leadership relies on the reward-punishment mechanism and may only intervene after workers make safety behavior mistakes, lacking proactive pre-event guidance, while supportive leadership can actively prevent the occurrence of safety problems in daily work (Bian et al., 2019; Faraz et al., 2018). Therefore, supportive leadership is particularly important in shaping the safety behaviors of new-generation construction workers. Although previous studies have demonstrated the significant impact of supportive leadership on employees’ attitudes and behaviors (Elsaied, 2019; Wei et al., 2013), limited research has explored the specific relationship between supportive leadership and the safety behaviors of the new generation of construction workers. Therefore, this study aims to investigate the underlying mechanisms through which supportive leadership influences the safety behaviors of this workforce.

From a theoretical standpoint, the supportive leadership style is grounded in social exchange theory (Cropanzano & Mitchell, 2005) and closely linked to both social cognitive theory (Bandura, 1999) and contingency theory (Kerr et al., 1974). This paper, therefore, investigates the relationship between supportive leadership and the safety behaviors of the new generation of construction workers within the context of construction safety, using social exchange theory, social cognitive theory, and contingency theory as its theoretical frameworks. Firstly, drawing from social exchange theory, in the context of complex construction environments with relatively high risks, supportive leaders proactively provide employees with both resources and emotional support, fostering a sense of care and respect. This approach helps employees attain psychological satisfaction and security, thereby motivating them to engage more actively in safety behaviors. Therefore, this study posits that supportive leadership positively influences the safety behaviors of the new generation of construction workers. Secondly, self-efficacy is a central concept in social cognitive theory. Individuals with high self-efficacy possess confidence in their ability to successfully complete tasks. When confronted with challenges in complex environments, they maintain a positive and optimistic outlook, driven by an internal force. They proactively employ effective strategies to regulate their behavior and performance, ensuring consistent progress toward their goals (Bandura, 1977, 1989). Empirical studies have demonstrated that different leadership styles significantly influence employees’ self-efficacy (Ren & Shen, 2024; Septriani, 2021; Zhen, 2020). Additionally, research across various fields has established that specific self-efficacy significantly impacts behavior and performance (Javed et al., 2021; Mo et al., 2023; F. Wang et al., 2022; Zhen, 2020). As Bandura (Bandura, 1977) noted, self-efficacy specific to a particular domain, task, or problem is more predictive of employees’ behaviors than general self-efficacy. Therefore, this study posits that, within the context of construction safety, safety self-efficacy may serve as a mediating variable between supportive leadership and the safety behaviors of the new generation of construction workers. Furthermore, contingency theory suggests that employees’ behaviors are influenced by the interaction between leadership styles and situational factors (Kerr et al., 1974). As a vital situational element, team safety climate has a significant moderating effect on the influence of various leadership styles on safety behaviors. (Kapp, 2012; Zhen, 2020). In the context of construction safety, the effect of supportive leadership on the safety behaviors of the new generation of construction workers is not static but varies depending on the level of team safety climate. Therefore, this study hypothesizes that team safety climate may moderate the influence of supportive leadership on both safety self-efficacy and safety behaviors among the new generation of construction workers.

In summary, this study makes several key contributions. First, it explores the relationship between supportive leadership and the safety behaviors of new-generation construction workers, adding valuable insights to the literature on leadership styles and behaviors. This research also advances both theoretical and empirical work in the field of behavioral science. Using frameworks such as social exchange theory, social cognitive theory, and contingency theory, the study unveils the internal mechanisms through which supportive leadership influences safety behaviors. Lastly, from a management perspective, this study offers effective recommendations and valuable guidance for enhancing the level of safety behaviors among new-generation construction workers.

The remaining six sections of the study are as follows: Section 2: theoretical basis and hypotheses; Section 3: research methodology; Section 4: research results; Section 5: discussion of the research results; Section 6: research constraints and future prospects; and Section 7: conclusions.

## 2. Theory Basis and Hypotheses

**Figure 1:**
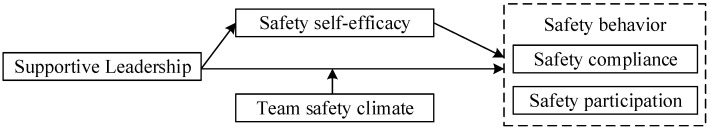
Model hypothesis.

This study adopted social exchange theory, social cognitive theory, and contingency theory to construct a research model (Figure 1), aiming to analyze the relationship between supportive leadership and the safety behaviors of new-generation construction workers.

### 2.1. Theoretical Basis

#### 2.1.1. Social Exchange Theory

The social exchange theory is regarded as a process of resource exchange (Cropanzano & Mitchell, 2005). The resources here include not only tangible substances such as money and gifts, but also intangible elements such as emotional support, praise, respect, and services. In terms of the nature of the exchange relationship, there exists a social exchange relationship between organizational members and the organization, which is similar to but goes beyond the scope of simple resource exchange. Members invest their time, energy, professional skills, and loyalty, expecting to obtain returns from the organization, such as financial rewards, career development opportunities, job satisfaction, respect, and recognition. From the perspective of the reciprocity principle (the core driving force of social exchange): When the organization provides care, resource support, or a favorable working environment for its members, the members will feel obliged to reciprocate the organization with higher performance, improved working methods, or additional efforts.

#### 2.1.2. Social Cognitive Theory

Social cognitive theory holds that employees’ behaviors are largely influenced by their cognitive processes (Bandura, 1999). In terms of individual behavior shaping, they form their own behavior patterns through observing and interpreting the organizational environment, work tasks, leadership styles, and the behaviors of colleagues. For example, new employees will observe how veteran employees communicate with their superiors and complete work tasks and then learn and imitate these behaviors. In terms of self-efficacy and performance, employees’ self-efficacy plays a crucial role in their work performance. When employees have a high level of self-efficacy regarding their ability to complete a certain work task, they will set more challenging goals and engage more actively in their work, striving to overcome difficulties. In terms of leader-employee interaction, the behaviors of leaders are important objects for employees to observe and learn from. If leaders demonstrate positive, responsible, fair, and problem-solving traits, employees will take these behaviors as examples and try to imitate them in their own work. For instance, when facing a project crisis, if the leader responds calmly and effectively coordinates resources to solve the problem, employees will also draw on the leader’s approach when encountering similar situations. Meanwhile, leaders’ evaluations and feedback to employees also affect employees’ self-efficacy. Positive feedback and encouragement can boost employees’ self-efficacy, making them more confident and dedicated at work.

#### 2.1.3. Contingency Theory

The contingency theory holds that many elements of an organization, such as leadership styles, structural designs, and incentive mechanisms, should be flexibly determined according to various variables in the internal and external environments of the organization (Kerr et al., 1974). From the perspective of organizational behavior, the contingency theory emphasizes that organizational behavior is the result of the interaction of multiple factors, and there is no one-size-fits-all optimal management approach. Leadership styles also need to be contingent upon situational factors. Fiedler’s contingency leadership model points out that the effectiveness of leadership depends on the degree of match between the leader’s style and situational factors (such as leader-member relations and team atmosphere). For example, in a situation where the leader-member relationship is good and there is a positive team atmosphere, a task-oriented leadership style will be more effective in motivating employees to complete tasks efficiently.

### 2.2. Research Hypotheses

#### 2.2.1. Supportive Leadership and Safety Behaviors

Safety behaviors encompass the actions exhibited by employees during the labor and production process that adhere to safety regulations and standards. These behaviors aim to safeguard both individual and collective safety, preventing the occurrence of accidents (X. Liu et al., 2015b). As a distinct form of work performance, safety behaviors reflect the overall effectiveness of an organization’s safety practices (Griffin & Neal, 2000). Therefore, research on the dimensions of safety behaviors should draw from studies on work performance. Locke et al. (1981) categorized work performance into two components: task performance and contextual performance. Building on this framework, Griffin and Neal (2000) extended these dimensions into the safety domain, categorizing safety behaviors into two behavioral dimensions: safety compliance and safety participation. Safety compliance refers to employees’ adherence to safety regulations, rules, operational procedures, and standards during the labor and production process. This includes actions such as wearing protective equipment, such as hard hats, when entering the construction site. In contrast, safety participation involves employees actively engaging in safety-related activities, such as participating in safety training sessions, studying the latest safety knowledge and skills, and collaborating with others. This may include sharing experiences, offering solutions to safety problems, and proactively contributing to safety management initiatives.

Social exchange theory suggests that human interactions are grounded in a series of reciprocal exchanges. When one party provides resources, support, and recognition to another, it typically expects corresponding returns. This reciprocal relationship significantly influences individuals’ behavioral performance (Cropanzano & Mitchell, 2005). Within the context of construction safety, the new generation of construction workers, with their distinct values and career aspirations, may respond positively to supportive leadership, which aligns well with their needs. Regarding safety compliance, the new generation places greater emphasis on humanistic care and respect in the work environment compared to the older generation. Research has shown that supportive leaders are effective at listening to workers’ concerns and improving working conditions, thereby fulfilling employees’ psychological contracts. This fosters a sense of obligation among workers to comply with safety regulations as a way of reciprocating the leaders’ trust and maintaining a positive work atmosphere. As a result, they are more likely to internally accept and consistently adhere to safety procedures, reducing the likelihood of safety violations (Banai & Reisel, 2007). Moreover, even in the face of complex and dynamic construction sites with high safety risks, a positive safety climate cultivated by supportive leaders can continually reinforce workers’ commitment to safety norms and stabilize their safety compliance behaviors (Jansen et al., 2016). When considering safety participation, the new generation of construction workers is motivated by a desire to demonstrate their value and engage in team-related activities. Supportive leaders provide resource support and encourage workers to offer suggestions on safety matters, thereby granting them the opportunity to contribute to safety management. This fosters a sense of reciprocity among workers, prompting them to actively participate in activities such as assisting with safety training and identifying potential hazards (Rafferty & Griffin, 2006; Sharma & Pearsall, 2016). Based on these observations, this study proposes the following research hypotheses:

**Hypothesis** **1a.** *Supportive leadership has a positive impact on the safety compliance of new-generation construction workers.*

**Hypothesis** **1b.** *Supportive leadership has a positive impact on the safety participation of new-generation construction workers.*

#### 2.2.2. The Mediating Effect of Safety Self-Efficacy

Self-efficacy is defined as “an individual’s belief in their capacity to apply their skills in order to accomplish particular tasks” (Bandura, 1999). As an essential part of social cognitive theory, self-efficacy can be divided into general self-efficacy and specific self-efficacy. Among these two types, specific self-efficacy has a more significant influence on molding an individual’s specific behaviors (Bandura, 1977). In the context of construction safety, safety self-efficacy is defined as “an individual’s confidence in their ability to perform safety-related tasks”. This concept differs from safety awareness by emphasizing personal confidence in executing safety tasks rather than just the knowledge of safety issues (Katz-Navon et al., 2007). Social cognitive theory posits that an individual’s self-efficacy is developed through ongoing interactions between employees and leaders, as well as through the observation of others’ behaviors (Pillai & Williams, 2004). In the context of construction work, leadership style functions as a guiding force, significantly shaping the new generation of construction workers’ perceptions and assessments of their own capabilities. This is particularly evident when the new generation of construction workers first arrives at the construction site, confronted by complex scaffolding and the loud operation of heavy machinery. At this stage, they often lack confidence in their ability to perform safety tasks effectively. At this stage, supportive leadership plays a critical role. This leadership style emphasizes providing subordinates with thoughtful care, constructive feedback, and ample resource support (K. Y. Kim et al., 2021). Specifically, supportive leaders organize and implement systematic and comprehensive safety training programs, offer practical guidance on the proper use of protective equipment on the construction site, impart valuable safety experience, and regularly review safety practices while recognizing employees’ safety-related performance. These actions help to strengthen workers’ confidence in safety management, facilitating a smooth transition from low to high safety self-efficacy. Therefore, the research hypotheses are proposed as follows:

**Hypothesis** **2.** *Supportive leadership has a positive impact on the safety self-efficacy of the new-generation construction workers.*

Safety self-efficacy functions as a powerful “engine”, driving the safety behaviors of new-generation construction workers. Antecedent research has demonstrated that self-efficacy significantly influences both employees’ behavioral intentions and their actual behaviors (Corbett, 2002; Rodgers et al., 2008). Specifically, safety self-efficacy is regarded as a key predictor of safety behaviors. A large number of empirical studies have demonstrated its positive role in promoting employees’ safety behaviors.

Specifically, safety self-efficacy is considered one of the key predictors of safety behaviors. Numerous empirical researches have demonstrated its positive impact in promoting safety behaviors among employees (Curcuruto et al., 2024; Jafari Nodoushan et al., 2024; F. Li & Han, 2020; Mo et al., 2023). For instance, studies have shown that workers with high safety self-efficacy are less likely to engage in safety violations than those with lower self-efficacy. They are also more likely to participate actively in safety-related volunteer activities and report potential hazards promptly (Ye et al., 2022). Based on these insights, this study proposes the following research hypotheses:

**Hypothesis** **3a.** *Safety self-efficacy has a positive impact on the safety compliance of the new-generation construction workers.*

**Hypothesis** **3b.** *Safety self-efficacy has a positive impact on the safety participation of the new-generation construction workers.*

In summary, safety self-efficacy serves as a “conduction bridge” between supportive leadership and the safety behaviors of new-generation construction workers—a relationship that has not been explored in previous studies. This study posits that supportive leadership, acting as an “external driving force”, indirectly influences the safety behaviors of these workers by reshaping their “psychological engine”, which is their safety self-efficacy. As previously discussed, supportive leadership is grounded in the principles of respect, understanding, and trust toward employees. It focuses on addressing employees’ work challenges and emotional needs, providing them with respect, care, and encouragement. This leadership style helps to stabilize employees’ confidence in safety control, thereby enhancing their safety self-efficacy. Moreover, employees with high safety self-efficacy believe they can employ effective strategies to manage their behaviors and mitigate potential negative outcomes, which ultimately leads to improved safety behaviors. Based on these insights, this study proposes the following hypotheses:

**Hypothesis** **4a.** *Safety self-efficacy plays a mediating effect between supportive leadership and the safety compliance of the new-generation construction workers.*

**Hypothesis** **4b.** *Safety self-efficacy plays a mediating effect between supportive leadership and the safety participation of the new-generation construction workers.*

#### 2.2.3. The Moderating Effect of Team Safety Climate

Team safety climate refers to the shared perceptions, attitudes, and feelings of team members regarding safety (Q. Li et al., 2017). From a cognitive perspective, it pertains to the extent to which employees understand safety regulations and operational standards. For instance, members of a construction team should be well-versed in the requirements for wearing safety protection equipment during high-altitude operations. In terms of attitude, it reflects the priority that team members place on safety issues. For example, when team members actively support safety inspections and proactively report safety hazards, it indicates a positive safety attitude within the team. At the level of feelings, it encompasses the members’ intuitive sense of safety within the team environment. For example, a team may feel confident working on a particular construction site, knowing that they are not at risk from others violating operational rules.

Contingency theory asserts that the effect of leadership styles on employee behaviors is not fixed but rather influenced by situational factors (Bandura, 1989). Previous studies have demonstrated that team safety climate, as a critical situational factor, plays a moderating role in the relationship between leadership styles and employees’ safety behaviors (Kapp, 2012; Zhen, 2020). However, in the context of construction safety, the supportive leadership style is characterized by the leader’s provision of resources to the new-generation construction workers, along with emotional care and encouragement. For instance, showing concern for employees’ work environment, fatigue levels, and addressing personal and safety-related challenges is essential. When the team safety climate is positive (refferring to an atmosphere with a high level of safety-related communication, shared safety values, and active support from management for safety measures), it functions as a robust bridge between the supportive leadership style and workers’ safety behaviors. In a construction team with a strong safety climate, where safety is prioritized and effective safety communication mechanisms and a positive safety culture are in place, the supportive actions of leaders—such as providing safety training and addressing workers’ difficulties with safety operations—are reinforced by this environment. This interaction encourages workers to become more proactive in learning safety knowledge and actively participating in activities such as assisting with safety training and identifying potential safety hazards. Conversely, when the team safety climate is poor (refferring to an atmosphere with sporadic and inefficient safety communication, a possible lack of shared safety values, and some members who will put short-term productivity before safety), the moderating effect of the contingency factor may be negative. For instance, in a construction team where safety awareness is weak and safety rules are frequently ignored, even supportive leadership may have a diminished impact on the safety behaviors of new-generation construction workers. For example, although the leader may emphasize the importance of safety equipment and provide new protective gear, the failure to correct noncompliance among other team members could lead the new-generation workers to perceive safety equipment as unnecessary, thereby reducing their motivation to engage in safety behaviors. In other words, the effectiveness of supportive leadership in promoting safety behaviors is contingent upon the prevailing team safety climate. Therefore, the research hypotheses are proposed as follows:

**Hypothesis** **5a.** *Team safety climate has a positive moderating effect between supportive leadership and the safety compliance of the new-generation construction workers.*

**Hypothesis** **5b.** *Team safety climate has a positive moderating effect between supportive leadership and the safety participation of the new-generation construction workers.*

## 3. Methods

This study adopts a combined approach of questionnaire surveys, confirmatory factor analysis, correlation tests, and linear regression. This approach can effectively examine the validity of the model, revealing the correlations and causal relationships among variables. These methods cooperate with each other, strongly supporting the reliability and scientific nature of the research conclusions.

### 3.1. Sample and Procedure

To mitigate the potential influence of common method bias, this study adhered to the established procedures outlined in previous research on construction workers’ safety behaviors and employed a staged data collection approach (Podsakoff et al., 2012; X. Wang et al., 2021). Prior to data collection, participants were informed about the research objectives, and their written consent was obtained to ensure confidentiality. During the questionnaire completion process, each participant was assigned a unique code to link their responses across different stages. Once the questionnaires from each stage were matched, the codes were subsequently removed to maintain data anonymity.

This study focuses primarily on the new-generation construction workers, gathering data through questionnaires. The data was collected from groups of new-generation construction workers across eight project departments in regions such as Gansu and Shaanxi. To ensure the accuracy of the responses and maximize the response rate, offline questionnaires were administered, with workers completing them during their off-duty hours. During the survey process, if participants encountered difficulties understanding any items, members of the research team were available to clarify and explain the questions that were perceived as ambiguous. The questionnaire-based investigation was implemented in a two-phase approach. During the initial phase, researchers gathered data on demographic variables, supportive leadership, and safety self-efficacy. After a two-month interval (to minimize the risk of response bias caused by participants’ habitual thinking), the second stage was carried out, focusing on team safety climate and safety behaviors. Upon completion of both stages, the two sets of questionnaires were combined, and any invalid responses, such as those filled out randomly, were excluded from the analysis.

The total sample size for this study was 332 participants. After excluding invalid responses, such as those filled out randomly, 285 valid questionnaires remained, yielding a response rate of 86%, which met the sample size requirements for the study (Junior, 1992). Of these valid responses, the proportion of males was 88.8% (n = 253), while that of females was 11.2% (n = 32). In terms of age distribution, 16.1% (n = 46) were aged 20–29, 58.2% (n = 166) were aged 30–39, and 25.6% (n = 73) were aged 40–49. Regarding work experience, 33.3% (n = 95) had 0–5 years of experience, 40.4% (n = 115) had 6–10 years, and 26.3% (n = 75) had more than 11 years of experience.

### 3.2. Measures

All the scales used in this study have passed consistency and reliability tests and are widely cited in empirical research. To ensure their accuracy and consistency, we used a translation and back-translation process to translate them from English to Chinese, confirming cultural suitability and semantic clarity. Each scale uses a seven-point Likert scale, with responses from 1 (strongly disagree) to 7 (strongly agree).

Supportive Leadership: This study utilized the eight-item scale developed by Rooney and Gottlieb (2007), which is widely recognized and frequently applied in research on supportive leadership. The scale has been extensively used in China and demonstrates strong reliability and validity (Prihandaka et al., 2022; Yang, 2017). An example item from the scale is: “My supervisor often provides help with difficulties in the lives of team members”.

Safety Self-Efficacy: We made use of the six-item general self-efficacy scale developed by Chen et al. (2001), with each item adapted to specifically assess safety self-efficacy. As previously noted, specific self-efficacy has a more pronounced effect on influencing an individual’s behavior in particular domains. Thus, the general self-efficacy scale was tailored to focus on the safety context in order to construct the safety self-efficacy scale. An example item from the safety self-efficacy scale is: “I will do my best to accomplish my work goals as long as they are set”.

Team Safety Climate: The team safety climate was measured by means of the four-item scale developed by Wu et al. (2017). One of the example items is: “If we encounter troubles, we will help each other”.

Safety Behavior: To assess safety behavior, this study adopted the seven-item scale developed by Griffin and Neal (2000), which includes two dimensions: safety compliance and safety participation. The safety participation dimension consists of three items, such as “I make extra efforts to improve safety in the workplace”. The safety compliance dimension contains four items, such as “I follow the correct safety procedures to complete my work”.

Control variables: Previous research indicates that demographic factors may influence construction workers’ safety behaviors (He et al., 2019; Mo et al., 2023). Specifically, older workers and those with more work experience tend to exhibit more safety behaviors. Therefore, this study includes gender, age, and work experience as control variables to account for their potential effects on safety behaviors.

## 4. Results

This study employed SPSS 24.0 (PROCESS procedure) and AMOS 24.0 (Hayes, 2012) for statistical analysis. The analysis was conducted in three steps. First, discriminant validity and common method bias were assessed. Second, descriptive statistics and correlation analysis were performed for each research variable, followed by tests of convergent validity and composite reliability. Finally, in the third step, hierarchical regression analysis was utilized to test the support for the proposed hypotheses.

### 4.1. Reliability and Consistency Testing

We performed reliability and consistency tests on all variables by utilizing SPSS 24.0 software. The results are presented in Table 1. For all the variables in this study, the Cronbach’s coefficients exceed 0.8, the composite reliability values (CR) are higher than 0.8, the average variance extracted values (AVE) are above 0.5, and the factor loadings are greater than 0.7 but less than 1. Consequently, all the variables in this study demonstrate excellent reliability and consistency.

### 4.2. Confirmatory Factor Analysis

We utilized SPSS 24.0 software to carry out a series of confirmatory factor analyses (CFA) to assess whether the model in this study possesses good discriminant validity. As presented in Table 2, the goodness-of-fit indices of all models were tested. Through comparison, it was found that the five-factor model (χ^2^ = 623.069, df = 265, 1 < χ^2^/df = 2.351 < 3, RMSEA = 0.069 < 0.08, CFI = 0.921 > 0.9, TLI = 0.911 > 0.9, SRMR = 0.044 < 0.08) exhibited good discriminant validity. Compared with other models, this model has the optimal goodness-of-fit. Therefore, the model adopted in this research exhibits excellent discriminant validity.

As the survey data were collected through self-reporting, and the key variables in each questionnaire were provided by the same respondents, there exists a potential for common method bias. To mitigate the potential impact of common method bias on this study, several strategies were adopted, such as utilizing anonymous, staged, and multi-source data collection methods. Moreover, SPSS 24.0 was utilized to carry out Harman’s single-factor test and factor analysis. The findings indicated the existence of five factors having eigenvalues greater than 1, and the largest factor was responsible for 31.67% of the variance, which was below the 40% threshold. This implied that common method bias does not have a substantial impact on the results of this study (S.-M. Liu et al., 2015a; Xiong et al., 2012).

### 4.3. Descriptive Statistics and Correlations

Table 3 exhibits the descriptive statistics and correlation analysis of the variables within this study. The results demonstrate that supportive leadership was positively correlated with safety self-efficacy (*r* = 0.268, *p* < 0.01), team safety climate (*r* = 0.130, *p* < 0.01), safety compliance (*r* = 0.549, *p* < 0.01), and safety participation (*r* = 0.336, *p* < 0.01). Safety self-efficacy was positively correlated with team safety climate (*r* = 0.147, *p* < 0.05), safety compliance (*r* = 0.463, *p* < 0.01), and safety participation (*r* = 0.325, *p* < 0.01). Furthermore, team safety climate was positively correlated with safety compliance (*r* = 0.133, *p* < 0.05) and safety participation (*r* = 0.184, *p* < 0.01). Finally, safety compliance (*r* = 0.463, *p* < 0.01) and safety participation (*r* = 0.227, *p* < 0.01) were also positively correlated with each other. These research results provide preliminary support for the hypothesized relationships among the variables.

### 4.4. Hypotheses Testing

First, this study conducted data analysis using SPSS and employed the PROCESS v3.3 macro to analyze the total effect, direct effect, and mediating effect (Hayes, 2012). The total effect of supportive leadership on safety behavior, as well as the direct and indirect effects of supportive leadership on safety behavior through safety self-efficacy, were tested. The results in Table 4 and Table 5 show that supportive leadership has a significant positive impact on safety compliance (*β* = 0.458, *t* = 10.881, *p* < 0.001) and safety participation (*β* = 0.307, *t* = 5.890, *p* < 0.001). When the mediating variable is included, supportive leadership still has a significant positive impact on safety compliance (*β* = 0.379, *t* = 9.370, *p* < 0.001) and safety participation (*β* = 0.245, *t* = 4.644, *p* < 0.001). Thus, hypotheses H1a and H1b are supported. Additionally, supportive leadership has a significant positive impact on safety self-efficacy (*β* = 0.277, *t* = 4.839, *p* < 0.001), thus supporting hypothesis H2. Safety self-efficacy also has a significant positive impact on safety compliance (*β* = 0.283, *t* = 6.973, *p* < 0.001) and safety participation (*β* = 0.226, *t* = 4.270, *p* < 0.001), thereby supporting hypotheses H3a and H3b. Moreover, the direct effects of supportive leadership on safety compliance and safety participation, as well as the mediating effect of safety self-efficacy, do not include 0 in the bootstrap 95% confidence interval (see Table 5), indicating that supportive leadership not only directly predicts safety behavior but also predicts safety behavior (safety compliance and safety participation) through the mediating effect of safety self-efficacy. Therefore, hypotheses H4a and H4b are supported.

**Figure 2:**
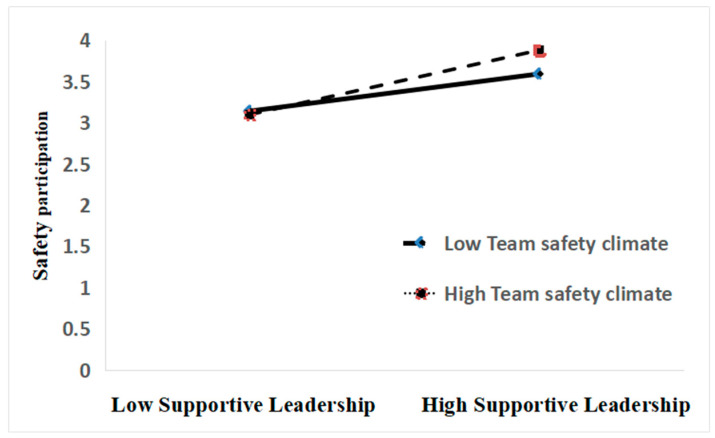
Interactive effect of supportive leadership and team safety climate on safety participation.

Next, a moderation effect analysis was conducted using the PROCESS v3.3 macro (Hayes, 2012). This analysis tested the moderating role of team safety climate in the relationship between supportive leadership and safety behavior (safety compliance and safety participation). The results (Table 6) show that when team safety climate is included in the model, the interaction term of supportive leadership and team safety climate has a significant positive effect on safety participation (*β* = 0.165, *t* = 2.694, *p* < 0.01) but does not have a significant positive effect on safety compliance (*β* = 0.002, *t* = 0.035, *p* > 0.05). This suggests that team safety climate has a positive moderating effect between supportive leadership and safety participation, thus supporting hypothesis H5b, while hypothesis H5a is rejected. Furthermore, simple slope analysis of the supported hypothesis H5b (Figure 2) shows that for participants with low team safety climate (L), supportive leadership does not have a significant impact on safety participation (*k* = 0.098, *t* = 1.326, *p* > 0.05), whereas for participants with high team safety climate (H), supportive leadership has a significant positive effect on safety participation (*k* = 0.353, *t* = 4.804, *p* < 0.001). This indicates that as the level of team safety climate increases, the predictive effect of supportive leadership on safety participation increases (see Table 5). In contrast, simple slope analysis of hypothesis H5a shows no significant difference in the effect of supportive leadership on safety participation between participants with low (L) and high (H) team safety climate, suggesting that team safety climate does not moderate the relationship between supportive leadership and safety participation.

## 5. Discussion

Based on the aforementioned theories, this study uncovers the internal operational mechanism by which supportive leadership impacts the safety behaviors of new-generation construction workers. Specifically, it not only elucidated how supportive leadership affects the safety behaviors of new-generation construction workers—via the mediating role of safety self-efficacy—but also identified the conditions under which this influence is most pronounced, through the moderating role of team safety climate. This research contributes to the literature by advancing the application of supportive leadership in the construction safety domain and offers both theoretical and practical insights into enhancing the safety behaviors of new-generation construction workers.

### 5.1. Theoretical Implications

This study has three major theoretical contributions to the literature.

Firstly, this study contributes to the growing body of research on the relationship between leadership styles and the safety behaviors of construction workers. It highlights the pivotal role of supportive leadership in fostering safety behaviors among new-generation construction workers. Specifically, the new-generation construction workers have been exposed to harsh working environments for a long time. Construction sites are filled with severe dust and noise pollution, as well as high risks of safety accidents, which constantly erode their physical and mental health. The high-intensity and long-hour working patterns deprive them of much-needed rest time, leaving them exhausted both physically and mentally. With limited career development prospects, they can hardly see hope for the future. Socially isolated and under tremendous financial pressure, they are trapped in multiple dilemmas and thus prone to developing negative psychological states such as anxiety and depression (Sámano-Ríos et al., 2019; Yu et al., 2022; Zhou et al., 2024). For example, employees afflicted by anxiety are likely to encounter difficulties in concentrating on their tasks, which may consequently lead to the inadvertent disregard of safety protocols. Simultaneously, employees suffering from depression may exhibit a lack of initiative in participating in activities aimed at enhancing safety, thereby contributing to an augmentation of accident risks. Previous research has predominantly focused on the influence of transformational leadership on employees’ safety behaviors (Hoffmeister et al., 2014; B.-J. Kim & Jung, 2019; D. Wang et al., 2023). Transformational leadership typically emphasizes creating an overarching organizational climate centered around safety goals, influencing employees’ safety behaviors primarily through the formulation of a compelling organizational vision and related strategies (D. Wang et al., 2023). In contrast, supportive leadership prioritizes employees’ well-being and job satisfaction. It compensates for employees’ lack of experience by offering guidance, providing resource support, and delivering emotional care to improve employees’ negative psychological conditions such as anxiety and depression. Through these mechanisms, supportive leadership helps employees recognize their value and feel respected in the workplace (Wei et al., 2013). Consistent with social exchange theory, which posits that high-quality exchange relationships foster positive employee behaviors (Cropanzano & Mitchell, 2005), this study further elucidates that when supportive leadership fosters a strong social relationship with new-generation construction workers, it promotes their perception of respect, understanding, care, and resource support from their leaders. This, in turn, stimulates their internal motivation for positive behavior, leading to enhanced safety behaviors (safety compliance and safety participation). Thus, this study not only provides a theoretical foundation for improving the safety behaviors of new-generation construction workers through optimized leadership styles but also offers a fresh perspective on the practical application of leadership styles in construction safety management.

Secondly, the social cognitive theory posits that individuals shape their behaviors through cognitive processing and self-regulation. A key element of this theory, self-efficacy, significantly influences individuals’ actions (Bandura, 1977, 1999). This study builds upon the work of Qin (2022), exploring the role of safety self-efficacy in the context of construction safety. Specifically, it examines the mediating effect of safety self-efficacy on the influence of supportive leadership on safety behaviors. Notably, this study represents the first attempt to uncover the “black box” linking supportive leadership to safety behaviors. Previous research has established that self-efficacy serves as a mediator between leadership styles and employee behaviors (Javed et al., 2021; Septriani, 2021). As expected, safety self-efficacy has a significant mediating effect between supportive leadership and the safety behaviors of the new-generation construction workers. This is consistent with the research results of Zhen (Javed et al., 2021; Septriani, 2021), that is, safety self-efficacy has a mediating role between leadership styles and employees’ safety behaviors. Moreover, our empirical results support the view of F. Li and Han (2020) that safety self-efficacy plays a decisive role in predicting safety behaviors. This research result expands the application breadth and depth of the social cognitive theory in the specific occupational safety field and provides an example for the specific application of self-efficacy in different industries in subsequent studies.

Thirdly, this study enriches the application of contingency theory, which emphasizes that the effectiveness of leadership depends on the degree of match between the leader’s style and situational factors (such as leader–member relations and team atmosphere) (Kerr et al., 1974). Previous research has demonstrated that team safety climate, as a critical situational factor, moderates the association between leadership styles and employee behaviors (Kapp, 2012; Zhen, 2020). This study extends this concept to the context of construction safety, specifically examining the moderating role of team safety climate in the relationship between supportive leadership and the safety behaviors (safety compliance and safety participation) of new-generation construction workers. The study found that team safety climate plays a significant positive moderating role in the relationship between supportive leadership and the safety participation of new-generation construction workers. Conversely, the team safety climate does not exert a notable moderating effect on the influence that supportive leadership style has on safety compliance behavior. This finding differs from the results of Kapp (2012), whose research indicated that team safety climate significantly moderates the relationship between transformational leadership and safety compliance among construction workers. The difference in findings can be attributed to the distinct nature of transformational leadership, which primarily motivates employees through the articulation of inspiring visions. In the context of construction safety management, transformational leaders convey compelling goals, such as “creating the safest construction site”, thereby elevating safety objectives to a higher level of importance. Within a positive team safety climate, communication flows more effectively, enabling team members to share and reinforce the safety vision promoted by transformational leadership. For instance, during daily operations, team members discuss how to achieve the goal of a “zero-accident construction site” by adhering to established safety protocols. Such communication reinforces the safety vision, making it more deeply ingrained in workers’ minds, thereby enhancing the importance construction workers place on safety compliance. In contrast, supportive leadership focuses primarily on addressing employees’ personal needs and offering emotional and practical support. For new-generation construction workers, this direct form of support may play a more prominent role in encouraging safety compliance. For instance, to ensure adherence to the safety rule of wearing helmets, the leader might provide detailed explanations on how helmets protect workers’ lives, supply comfortable and high-quality helmets, and consistently remind and supervise workers to wear them throughout the workday. This kind of direct guidance and attention could lead new-generation construction workers to prioritize the immediate instructions and support from their leaders, thereby diminishing the moderating effect of the team safety climate. Additionally, this study found that the impact of supportive leadership on safety compliance was significantly greater than on safety participation. Therefore, the findings of this research are consistent and plausible. This study expands the scope of contingency theory within safety management by exploring the interplay between different behavioral outcomes, leadership styles, and situational factors, contributing to a deeper understanding of the differentiated mechanisms through which contingency factors influence safety behaviors.

### 5.2. Practical Implications

This research presents three primary practical implications.

Firstly, the research findings highlight the positive impact of supportive leadership on the safety behaviors of new-generation construction workers. Therefore, construction companies should prioritize the development of supportive leadership traits and competencies when selecting and training leaders. For current leaders, specialized training workshops focusing on supportive leadership can be implemented. These workshops could utilize methods such as case analysis and role-playing to enhance leaders’ ability to perceive and respond to the needs of new-generation construction workers. Additionally, when selecting new leaders, it is essential to not only consider professional qualifications and management experience but also evaluate whether candidates exhibit a supportive leadership style as part of the selection criteria. For example, evaluating candidates’ past performance in areas such as motivating subordinates, providing necessary resources, and offering emotional support can help ensure that newly appointed leaders actively promote safety behaviors among workers. In addition, creating efficient two-way communication channels is crucial to facilitating the smooth exchange of information between leaders and workers (Lingard et al., 2019). Leaders should regularly organize group discussions or one-on-one exchanges focused on safety issues, encouraging workers to share potential safety hazards, challenges in their work, and their perspectives on safety management measures. At the same time, leaders must clearly and promptly communicate the company’s safety policies, goals, and requirements. An effective feedback mechanism should also be established to ensure that workers’ safety suggestions and concerns are addressed promptly. The outcomes of these actions should be communicated back to the workers, allowing them to feel that their voices are heard and valued. This, in turn, will strengthen their trust in leadership and motivate them to actively adhere to safety regulations and participate in safety management efforts.

Secondly, the research highlights the mediating role of safety self-efficacy in the relationship between supportive leadership and the safety behaviors of new-generation construction workers. To enhance this effect, supportive leaders should prioritize strengthening the safety knowledge and skills training system for these workers. It is crucial to ensure that the training content is both comprehensive and practical, covering essential areas such as construction safety regulations, hazard identification, and response strategies. Additionally, a variety of training methods should be employed, including on-site demonstrations, multimedia presentations, and interactive group discussions, to improve workers’ understanding and retention of safety knowledge. Through such systematic training, workers can more effectively apply safety skills in real-world scenarios, thereby boosting their safety self-efficacy and promoting the enactment of safety behaviors. Furthermore, leaders should actively encourage workers to participate in safety-related decision-making and management processes. For instance, when developing on-site construction safety systems and measures, it is essential to actively seek input from new-generation construction workers. By involving them in the decision-making process, workers will feel a sense of influence and responsibility in safety management, which enhances their self-confidence and safety self-efficacy. This, in turn, encourages greater proactivity in executing safety tasks. Additionally, establishing clear safety goals and a well-structured reward system is crucial. Large safety objectives should be broken down into smaller, measurable, and achievable targets at various stages. Upon meeting these targets, workers should receive timely recognition and rewards, whether material or symbolic. Such incentives not only reinforce their belief in their safety capabilities but also further bolster their safety self-efficacy, ultimately contributing to improved overall safety behaviors.

Thirdly, the research highlights the significant role of the interaction between supportive leadership and team safety climate in influencing the safety behaviors of new-generation construction workers, particularly the moderating effect of team safety climate on the relationship between supportive leadership and these safety behaviors. To leverage this dynamic, leaders should actively cultivate a positive team safety climate. This includes clearly communicating safety standards and regulations on the construction site and enhancing awareness through various means, such as posting prominent safety slogans and organizing regular safety training sessions and knowledge lectures. These efforts help embed a safety-conscious mindset among workers (Liyanagamage, 2024). Moreover, leaders should model safety behaviors by strictly adhering to safety protocols themselves, thereby setting an example for others to follow. This helps integrate safety as a core component of the team culture. Additionally, leaders should foster safety-related interaction and collaboration among team members. This can be achieved by organizing safety exchange activities, such as safety experience sharing sessions and safety hazard inspection groups. Encouraging new-generation construction workers to actively participate in these activities allows them to share their safety experiences, learn from each other, and collaboratively address safety challenges. Through such interactions, team cohesion and members’ sense of belonging are strengthened, thereby enhancing the team safety climate and motivating workers to engage more actively in safety management.

## 6. Research Constraints and Future Prospects

Even though comprehensive and strenuous attempts have been made to protect the objectivity and scientific exactitude of this research, it is crucial to be cognizant of and concede that there are a number of constraints. Firstly, due to constraints in time, budget, and other factors, the data collection for this study was primarily focused on construction projects in specific regions. As a result, the regional representativeness of the sample was limited, which may have hindered the ability to fully capture the diverse characteristics and variations within the construction industry across different regions. This limitation could impact the generalizability of the study’s findings. Therefore, future research should consider expanding the sample to include new-generation construction workers from construction enterprises of varying sizes and across different geographic locations. Such an approach would help to enhance the representativeness and broader applicability of the research outcomes. Future research should focus on further optimizing measurement tools by developing more accurate and effective scales for assessing relevant variables or by combining multiple measurement methods to enable cross-validation. Longitudinal research designs or experimental methods could be employed to better establish causal relationships among the variables and to gain deeper insights into the mechanisms through which supportive leadership influences the safety behaviors of new-generation construction workers. Additionally, this study primarily considered the mediating role of safety self-efficacy and the moderating role of team safety climate. Future studies could expand this framework by exploring other potential mediators and moderators, such as individual safety cognition and safety culture. This would enhance the understanding of the complex relationships at play, thereby offering a more comprehensive theoretical foundation and practical guidance for improving safety management within the construction industry.

## 7. Conclusions

Grounded in social exchange theory, social cognitive theory, and contingency theory, this study elucidates the mechanisms through which supportive leadership influences the safety behaviors of new-generation construction workers. The findings indicate that supportive leadership has a significant positive impact on the safety behaviors of these workers, with its effect on safety compliance being more pronounced than its impact on safety participation. Moreover, safety self-efficacy plays a significant mediating role in the relationship between supportive leadership and safety behaviors, suggesting that supportive leadership indirectly influences the safety behaviors of new-generation construction workers by enhancing their safety self-efficacy. Moreover, an analysis of effect proportions reveals that the direct impact of supportive leadership on the safety compliance (82.9%) and safety participation (79.7%) of new-generation construction workers exceeds the mediating effect of safety self-efficacy in these relationships (17.1% for safety compliance and 20.3% for safety participation). Additionally, the research findings indicate that team safety climate plays a significant positive moderating role in the relationship between supportive leadership and safety participation. Specifically, the impact of supportive leadership on the safety participation of new-generation construction workers differs significantly depending on whether the team safety climate is perceived as high or low. To enhance the safety behaviors of new-generation construction workers, it is essential to not only foster the development of supportive leadership, ensuring that leaders effectively provide workers with adequate work resources, timely emotional support, and clear career encouragement—thereby boosting safety self-efficacy—but also to cultivate a positive team safety climate. The synergistic effect of supportive leadership and a favorable team safety climate is crucial for fully activating the internal motivation of new-generation construction workers. When these factors align, workers are more likely to consistently adhere to safety regulations throughout the construction process, safeguarding both their well-being and the project’s overall safety. This, in turn, can elevate the safety management standards within the construction industry.

## Figures and Tables

**Table 1:** Reliability tests, validity tests, and factor loading tests.

	Cronbach’s Coefficients	Composite Reliability	Average Variance Extracted	Factor Loadings
Supportive Leadership	0.921	0.924	0.602	0.745~0.816
Safety Self-Efficacy	0.883	0.896	0.590	0.703~0.831
Team Safety Climate	0.945	0.956	0.845	0.905~0.928
Safety Behavior (safety compliance)	0.860	0.835	0.560	0.764~0.783
Safety Behavior (safety participation)	0.890	0.842	0.640	0.766~0.817

**Table 2:** Model goodness-of-fit test.

Model	χ^2^	df	χ^2^/df	CFI	TLI	RMSEA	SRMR
Five-factor model	623.069	265	2.351	0.921	0.911	0.069	0.044
Four-factor model 1	1376.255	269	5.116	0.756	0.726	0.120	0.122
Four-factor model 2	1693.478	269	6.295	0.686	0.649	0.137	0.120
Three-factor model 1	1912.243	272	7.030	0.638	0.601	0.146	0.130
Three-factor model 2	2024.156	272	7.442	0.613	0.573	0.151	0.135
Two-factor model 1	2236.724	274	8.163	0.567	0.526	0.159	0.143
Two-factor model 2	2675.542	274	9.765	0.470	0.420	0.176	0.173
One-factor model	2886.430	275	10.496	0.424	0.371	0.183	0.167

Note: χ^2^ = chi-square; df = degree of freedom; four-factor model 1 = supportive leadership and safety self-efficacy combined; four-factor model 2 = supportive leadership and team safety climate combined; three-factor mode l = supportive leadership, team safety climate, and safety participation combined; three-factor mode 2 = supportive leadership, team safety climate, and safety compliance combined; two-factor mode 1 = supportive leadership, team safety climate, safety compliance, and safety participation combined; two-factor mode 2 = supportive leadership, safety self-efficacy, and team safety climate combined; safety compliance and safety participation combined.

**Table 3:** Descriptive statistics and correlation coefficients for variables.

Variable	1	2	3	4	5	6	7	8
1. Gender	/							
2. Age	0.399 **	/						
3. Work experience	0.242 **	0.513 **	/					
4. Supportive leadership	0.024	0.085	0.003	**/**				
5. Safety self-efficacy	0.003	0.120 *	0.215 **	0.268 **	**/**			
6. Team safety climate	0.054	0.076	0.142 *	0.130 *	0.147 *	**/**		
7. Safety compliance	0.037	0.135 *	0.079	0.549 **	0.463 **	0.133 *	**/**	
8. Safety participation	0.074	0.109	0.123 *	0.336 **	0.325 **	0.184 **	0.227 **	**/**
Mean	1.110	2.090	1.930	3.761	4.712	3.258	4.797	4.180
SD	0.316	0.640	0.770	0.819	0.832	0.777	0.690	0.765

Note: * represents *p* < 0.05, ** represents *p <* 0.01.

**Table 4:** Tests of total effect, direct effect, and mediating effect.

Variables	Total Effects				Direct and Indirect Effects						Moderating Effects			
	Safety Compliance		Safety Participation		Safety Self-Efficacy		Safety Compliance		Safety Participation		Safety Compliance		Safety Participation	
	*β*	*t*	*β*	*t*	*β*	*t*	*β*	*t*	*β*	*t*	*β*	*t*	*β*	*t*
Gender	0.025	0.175	−0.279	−1.566	0.265	1.356	−0.050	−0.375	−0.339	−1.954	−0.024	−0.178	−0.373	−2.175
Age	0.064	0.797	0.128	1.279	−0.116	−1.057	0.097	1.300	0.154	1.584	0.087	1.165	0.183	1.908
Work experience	0.044	0.703	0.039	0.497	0.307	3.576	−0.043	−0.714	−0.030	−0.392	−0.027	−0.448	−0.066	−0.858
Supportive leadership	0.458	10.881 ***	0.307	5.890 ***	0.277	4.839 ***	0.379	9.370 ***	0.245	4.644 ***	0.387	9.501 ***	0.225	4.321 ***
Safety self-efficacy							0.283	6.973 ***	0.226	4.270 ***	0.289	7.068 ***	0.205	3.912 ***
Team safety climate											0.071	1.683	0.123	2.284 **
interactive											0.002	0.035	0.165	2.694 **
R	0.557		0.368		0.352		0.642		0.434		0.647		0.472	
R^2^	0.310		0.135		0.124		0.413		0.189		0.419		0.223	
F	31.498 ***		10.970 ***		9.865 ***		39.211 ***		12.963 ***		28.497 ***		11.359 ***	

Note: ** represents *p* < 0.01, and *** represents *p* < 0.001; Interactive = Supportive leadership × Team safety climate.

**Table 5:** Decomposition of direct effect and indirect effect.

Path	Effect	SE	Bootstrapping 95% CI	
			LL 95% CI	UL 95% CI
Supportive leadership → Safety compliance	0.379	0.041	0.300	0.459
Supportive leadership → Safety self-efficacy → Safety compliance	0.078	0.020	0.043	0.119
Supportive leadership → Safety participation	0.245	0.053	0.141	0.349
Supportive leadership → Safety self-efficacy → Safety participation	0.063	0.020	0.029	0.107

Note: LL = lower limit; UL = upper limit; and CI = confidence interval.

**Table 6:** Decomposition of moderating effect.

Moderating Variables	Path: Supportive Leadership → Safety Compliance			Path: Supportive Leadership → Safety Participation		
	Effect	Bootstrapping 95% CI		Effect	Bootstrapping 95% CI	
		LL 95% CI	UL 95% CI		LL 95% CI	UL 95% CI
(H) High-Level team safety climate	0.386	0.275	0.496	0.097	−0.045	0.239
(L) Low-Level team safety climate	0.388	0.282	0.494	0.353	0.218	0.489

Note: LL = lower limit; UL = upper limit; M = Mean; and CI = confidence interval.

## Data Availability

Due to the ongoing nature of the research and analysis, the datasets generated and/or analyzed throughout the present study are not made publicly accessible at this time. Nevertheless, upon a reasonable request, these datasets can be obtained from the corresponding author.
